# Standards in Genomic Sciences

**DOI:** 10.4056/sigs.34251

**Published:** 2009-07-20

Late in the evening, forty years ago on this day, the attention of the world was focused on the actions of two men, who were the first to set foot on the moon.  It was largely a symbolic event, demonstrating a proof of principle and the collective accomplishment of thousands of scientists, engineers, and supporting personnel working towards a collective, albeit highly competitive technological goal.  It was a demonstration of potential of what could be achieved and what lay ahead rather than an end unto itself. In the ensuing years, there have been countless other achievements in national and international space programs that are of much greater scientific and technical significance, but received far less attention by the general public. Regardless of how remarkable each new discovery or achievement was, space exploration had become routine. 

The public announcement of the completion of the first genome sequence was no less of a remarkable collective, technological accomplishment.  While the size of the viewing audience for that event paled in comparison to the moon landing, it was no less in significance as an indicator of the potential importance that genome sequencing would have in the future.  Within a very brief time, we would pass the 100-sequenced genome milepost, then 1000-sequenced genome mark.  Like space exploration, genome sequencing became routine and the attention grabbing value of the announcement of another sequenced genome diminished. 

Now, the real challenge lies in the systematic exploration of the rapidly accumulating data and extraction of new information, insights and knowledge from each new genome sequence as it is added to the others that precede it.  Planting a flag and laying a claim is but the first step in exploration of new territory.  It is the task of the surveyors and cartographers to carefully map out the new territory, to set benchmarks, to identify landmarks and to establish ground truth.  An analogous task lies ahead for computational biologists, bioinformaticians, and annotators as they begin to map out biological space as it is defined by genome sequence data.  Those data will be critical for establishing biological ground truth across a vast phylogenetic space and essential for framing past, present and future biological knowledge.  

Cataloging and maintaining that information in an orderly, and standardized manner will be critical if it is to retain any value in the future.  Having publically available genome sequence data is useful. Having publically available genome sequence data that can be placed into a meaningful context (e.g. biologically, ecologically, geographically) and is linked to the biological literature is considerably more useful, especially if that information is readily available and accessible by a variety of routes.  That is a long-term goal of the *Genomic Standards Consortium*, which has been working to develop community standards for descriptive ‘omics metadata since 2005.  

As an open access publication, *Standards in Genomic Sciences* aims to fill the void that has been developing for the past few years.  As we pointed out previously, the rate at which new “genome papers” are appearing in the scholarly literature has plummeted at the very point in time when such papers are likely to become increasingly important as benchmarks that can be referenced repeatedly in other papers.  We aim to address this with the rapid publication of “Short Genome Reports” that are created in a highly structured form. The articles are designed so that they are easy to read and glean relevant facts about the source organism, the sequencing methodology and the annotation methodology.  Each article is accompanied by a supplementary table of MIGS compliant contextual data, based on the standard we defined in 2008.  

In this issue, we include seven Short Genome Reports for previously unpublished genome sequences that are part of the collaborative *Genomic Encyclopedia of Bacteria and Archaea* (GEBA) project between the US Department of Energy Joint Genome Institute (DOE-JGI) and the German National Culture Collection (DSMZ).  The source organisms included in this project are all taxonomic types and serve as name-bearing benchmarks that essential for delineation of the phylogenetic space occupied by *Bacteria* and *Archaea*. We believe that the incorporation of rich standardized biological information along with links into the taxonomic and genomic literature will provide readers with a unique, multi-faceted view of the data.

In addition to the Short Genome Reports, *SIGS* will also publish articles that we believe are of importance to the broad ‘omics community. These include Meeting Reports that provide summaries of the output and collective views of established working groups and other panels that are assembled by various agencies and communities of like-minded individuals to address problems of concern or explore new opportunities that lie on the near or far horizon. Two meeting reports are included in this issue that provide readers with summaries of the most recent meetings of the *Genomic Standards Consortium* in the fall of 2008 and the first meeting of the *SIGS* editorial and advisory boards in March, 2009.

Also included in this issue of *SIGS* are two white papers that are thought to be of interest to the community.  One is an executive summary of a much larger report that was commissioned by the Secretariat of the UN Convention on Biological Diversity (CBD) addressing issues of importance to negotiator: the use of DNA based methods for monitoring and tracking genetic resources under the terms of the treaty and how persistent identifiers can be used to link together data and metadata associated with mandatory records of various types of transactions involving genetic resources under the terms of the CBD. While this article was written for a very broad audience of scientists and non-scientists, it is included in *SIGS* because the CBD is likely to have very far-reaching ramifications.  It will affect genomics and it is important that this community have a voice in the process. The second whitepaper is a description of the recently funded NSF Research Center Network, that will help the *Genomic Standards Consortium* reach its next goals for extending ‘omics standards for contextual metadata more broadly (to wider communities using ‘omics data for a variety of purposes) and deeply (to incorporate an increasing rich set of contextual data descriptors and associated data with genomic data).

Of course, starting a new journal is not an inexpensive proposition, either in terms of labor or start-up costs.  We are grateful to the Vice President of Research and Graduate Studies at Michigan State University, the Michigan State University Foundation for financial support, and the Department of Microbiology and Molecular Genetics, Michigan State University for hosting the editorial office of SIGS. We are also grateful for the support provided by the US Department of Energy Office of Biological and Environmental Sciences to underwrite the first meeting of the SIGS editorial and advisory boards.  Lastly, we are grateful to the members of the editorial and advisory boards for their time and expertise, and to the many authors and reviewers of the articles appearing in this and all subsequent issues of SIGS.

On behalf the editorial board of *Standards in Genomic Sciences* and the *Genomic Standards Consortium*, we would like to welcome you as readers, authors and reviewers of the journal.  Comments are welcome and should be directed to editorial@standardsingenomics.org

George Garrity, 

Dawn Field,

Nikos Kyrpides

July, 20, 2009

